# Deep-Learning-Based AI-Model for Predicting Dental Plaque in the Young Permanent Teeth of Children Aged 8–13 Years

**DOI:** 10.3390/children12040475

**Published:** 2025-04-07

**Authors:** Banu Çiçek Tez, Yasin Güzel, Bahar Başak Kızıltan Eliaçık, Zafer Aydın

**Affiliations:** 1 Department of Pediatric Dentistry, Faculty of Dentistry, Ankara Medipol University, Ankara 06050, Türkiye; banu.tez@ankaramedipol.edu.tr; 2 Department of Educational Sciences, Suleyman Demirel University, Isparta 32200, Türkiye; 3 Department of Electrical and Computer Engineering, Abdullah Gul University, Kayseri 38080, Türkiye; zafer.aydin@agu.edu.tr; 4 Department of Pediatric Dentistry, Faculty of Dentistry, University of Health Sciences, Istanbul 34668, Türkiye; basak.eliacik@sbu.edu.tr

**Keywords:** AI models, deep learning, dental plaque, pediatric dentistry, health

## Abstract

Background/Objectives: Dental plaque is a significant contributor to various prevalent oral health conditions, including caries, gingivitis, and periodontitis. Consequently, its detection and management are of paramount importance for maintaining oral health. Manual plaque assessment is time-consuming, error-prone, and particularly challenging in uncooperative pediatric patients. These limitations have encouraged researchers to seek faster, more reliable methods. Accordingly, this study aims to develop a deep learning model for detecting and segmenting plaque in young permanent teeth and to evaluate its diagnostic precision. Methods: The dataset comprises 506 dental images from 31 patients aged between 8 and 13 years. Six state-of-the-art models were trained and evaluated using this dataset. The U-Net Transformer model, which yielded the best performance, was further compared against three experienced pediatric dentists for clinical feasibility using 35 randomly selected images from the test set. The clinical trial was registered on under the ID NCT06603233 (1 June 2023). Results: The Intersection over Union (IoU) score of the U-Net Transformer on the test set was measured as 0.7845, and the *p*-values obtained from the three *t*-tests conducted for comparison with dentists were found to be below 0.05. Compared with three experienced pediatric dentists, the deep learning model exhibited clinically superior performance in the detection and segmentation of dental plaque in young permanent teeth. Conclusions: This finding highlights the potential of AI-driven technologies in enhancing the accuracy and reliability of dental plaque detection and segmentation in pediatric dentistry.

## 1. Introduction

Dental plaque is defined as a microbial community embedded in a matrix composed of polymers derived from bacteria and the content of saliva that develops on the surface of the teeth [1]. Microbial dental plaque is adsorbed onto the tooth surface within seconds after dental cleaning and persists functionally [2]. These molecules primarily exist in the fluid of the subgingival sulcus, along with saliva, and demonstrate settlement in this area [3,4]. The primary etiological factor for gingivitis and periodontitis is bacterial plaque, which can lead to the destruction of gingival tissues and periodontal attachment [5]. In children, inadequate oral hygiene following tooth eruption and the absence of regular brushing habits facilitate the accumulation of bacterial biofilm on tooth surfaces and gingival margins. This accumulation, in turn, triggers gingival inflammation and increases the risk of periodontal disease [6].

The early detection and treatment of periodontal diseases in children are clinically crucial, as these conditions can progress and lead to adverse long-term outcomes [7]. Bacterial plaque is the primary etiological factor in gingival diseases among pediatric patients. However, identifying and distinguishing microbial dental plaque can be challenging for patients. Plaque detection is commonly performed in routine clinical practice using periodontal probes and/or plaque-disclosing solutions. While these methods are widely utilized, they may produce subjective results [8]. Moreover, such assessment techniques can be cumbersome, time-consuming, and ineffective, particularly in uncooperative children. Additionally, plaque-disclosing solutions may cause the temporary staining of the oral mucosa and lips, which can be a disadvantage in clinical practice.

Several plaque assessment indices, such as the Plaque Index [9] and the Quigley–Hein Index [10], are widely employed to evaluate dental plaque accumulation. However, these indices rely on subjective evaluations, limiting their consistency and reproducibility. In contrast, artificial intelligence (AI)-based models offer a more objective and automated approach to plaque detection. Manual plaque assessment is not only time-intensive but also prone to human error, particularly in high-volume clinical settings.

To address these challenges, digital technologies, including intraoral scanners and fluorescence-based techniques, have been explored for three-dimensional plaque detection. Additionally, advanced digital imaging methods, such as laser-induced autofluorescence spectroscopy and HIS color space analysis, have been investigated for microbial dental plaque detection. However, the widespread adoption of these techniques is hindered by limitations such as high equipment costs and the need for technical standardization [11,12].

For these reasons, this study aims to develop an affordable and easily accessible AI model for the early and accurate diagnosis of microbial dental plaque in children. The aim is to prevent various periodontal problems and provide motivation for oral hygiene by evaluating the diagnostic and detection performance of this AI model.

With advancements in AI and image processing, research on plaque detection, segmentation, and quantification in dental camera images has gained momentum [13,14,15,16,17,18,19,20,21,22,23]. However, despite these advancements, plaque detection and segmentation using AI have not yet become a gold standard. This has encouraged researchers to conduct further studies in this field. Therefore, this study aims to propose an AI model for predicting dental plaque in young permanent teeth of children. Finally, to evaluate the clinical feasibility of the model, statistical hypothesis tests are conducted to compare its predictions with assessments made by three experienced dentists.

## 2. Materials and Methods

In this study, a privately collected dataset was used to train six state-of-the-art AI models, incorporating variations of the U-Net architecture, for the prediction of dental plaque in the young permanent teeth of children. The performance of these models was then systematically evaluated and comprehensively analyzed. Finally, to assess the clinical applicability of the best-performing model, statistical hypothesis tests were conducted to compare its predictions with the assessments of three experienced dentists.

### 2.1. Dataset Collection

During the five-month data collection period, 31 pediatric patients aged 8 to 13 years, in the mixed dentition phase, were included in the study based on predefined inclusion criteria. These patients presented at the Pediatric Dentistry Clinic of Hamidiye Faculty of Dental Medicine, University of Health Sciences, and were randomly selected from those attending routine check-up appointments at a public hospital. Importantly, participants were not previously informed about the study and had not received any oral or dental hygiene education or motivational training prior to their participation.

Exclusion criteria included anterior young permanent teeth with enamel tissue integrity disruptions, such as decay, hypoplasia, and hypomineralization, as well as restored or prosthetically treated teeth. Additionally, young permanent teeth in the posterior region and primary teeth were not included in the study (see Table 1).

This study was approved by the Scientific Research Ethics Committee of Hamidiye at the University of Health Sciences and informed consent was obtained from the children’s legal guardians. The official trial protocol is publicly available on ClinicalTrials.gov under the ID NCT06603233.

After the teeth were isolated from saliva using cotton rolls, a saliva ejector, and an air spray, their initial photographs were captured using an intraoral camera (1280 × 720 pixels, TPC Ligang, China). To eliminate a deficiency in the study of You et al., images of the relevant tooth taken from different angles were also included in the dataset [18]. Subsequently, to reveal dental plaque, patients were instructed to chew a dental plaque-disclosing tablet (TePe-PlaqSearch™, Malmö, Sweden), which changes to a pink color, making the presence of plaque visible. Following this, the teeth were photographed for the second time from the same angles as the initial images.

The dataset used in this study was developed based on the O’Leary Plaque Score Index, a widely recognized and standardized method for assessing dental plaque accumulation [24]. To ensure consistency and reliability, all plaque annotations were performed following this index’s criteria. Plaque-disclosing agents were applied to the dental surfaces, and plaque presence was evaluated on three surfaces per tooth (mesial, distal, and buccal). Based on this assessment, the dataset was labeled in a binary manner as “plaque present” or “plaque absent”. These labeled images were then used to train the deep learning model, ensuring that it was developed using clinically validated ground truth data.

The photographs were cropped to ensure that only one complete tooth appeared in each image. After that, the plaque on the teeth in the initial photographs was annotated using the Visual Geometry Group (VGG) Image Annotator tool (version 2.0.11) by a specialized dentist based on the second set of photographs where the dental plaque was revealed [25]. The ground truth masks generated from the output of the VGG tool were used as labels during the training of deep learning models. Finally, the study included 506 photographs involving young permanent anterior teeth with corresponding ground truth masks.

The dataset is randomly divided into three sets: ~70% training (354 images), ~15% validation (73 images), and ~15% test (79 images). This division follows a tooth-based approach to ensure that images captured from different angles of the same tooth are included within the same subset. The 70-15-15 split is chosen as it provides an optimal balance for small to medium-sized datasets, allowing the model to learn effectively while ensuring sufficient data for validation and testing without compromising generalization.

### 2.2. The Architecture of Deep Learning Models

As artificial intelligence models, DeepLabV3+, Mask R-CNN (Detectron2), YOLOv8, UNet, Super Vision UNet and UNet Transformer models, which are state-of-the-art in semantic segmentation, were selected [26,27,28,29,30,31]. These models were implemented using the Python (version 3.10) programming language with the TensorFlow (version 2.11) and PyTorch (version 1.12) libraries. Due to the insufficient size of the training set containing 354 images for the training of these 6 models, data augmentation techniques were employed over the training dataset during training to increase this number.

Hyperparameter optimization involves identifying the optimal values for parameters that remain fixed during training and are not learned by the model itself, such as the learning rate, input size, and batch size. Proper hyperparameter tuning plays a crucial role in enhancing model performance, accuracy, and generalization ability, ensuring a more efficient and effective training process. For hyperparameter optimization, a grid search method was employed to tune the input image size (128, 192, and 256), batch size (2, 4, and 8), optimization algorithm (Adam, SGD, and RMSProp), and learning rate ($1\times 10^{-2}$, $1\times 10^{-4}$, $1\times 10^{-6}$) based on the model’s performance on the validation dataset [28,29,30]. The Detectron2 framework was used to implement the Mask R-CNN. However, since Detectron2 does not allow modifications to the optimization algorithm, SGD was used as the default optimizer. Additionally, the baseline models in Detectron2 (R101-C4, R50-C4, R50-DC5, and R50-FPN) were also optimized as part of the hyperparameter tuning process. The training epochs and the patience parameter for early stopping were uniformly set to 120 and 15, respectively, across all models [32,33,34].

### 2.3. Evaluation Metrics for Image Segmentation

Precision–recall [35], Intersection over Union (IoU) [36], and Dice Coefficient [37] are crucial metrics for evaluating the accuracy of image segmentation. Precision measures how many of the predicted positive regions are actually correct, helping to control false positives. Recall assesses the model’s ability to correctly identify all relevant regions, minimizing false negatives. The Dice Coefficient balances both precision and recall, providing a comprehensive measure of segmentation performance, while IoU directly quantifies the overlap between the predicted and actual segmented areas. The IoU score, which computes the ratio between the intersection and the union of two sets, is commonly used to evaluate the accuracy of prediction on semantic segmentation. These four metrics were used as metrics to evaluate the models in this study.

### 2.4. Statistical Analysis of the Difference Between the AI Model and Dentists

**Figure 1:**
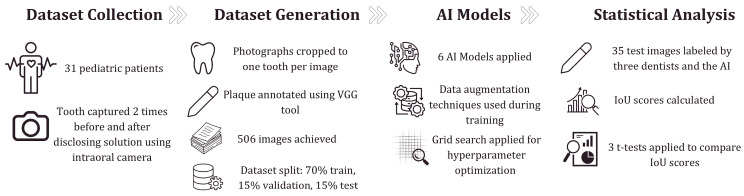
The workflow diagram of the study.

Using the prior knowledge (α = 0.05, β = 0.2) and an effect size of 0.61, the actual power of the comparison between the AI model and dentists on 34 test images is at least 80%, which is deemed sufficient. Therefore, 35 randomly selected images on the test dataset were labeled by three dentists without seeing the ground truth and were predicted by the AI model. Then, the IoU score of these labeled and predicted images were calculated. To confirm clinical feasibility, three *t*-tests, which evaluates the difference between the means of two variables, were applied to IoU scores of dentists and IoU scores of the AI model and a *p* value < 0.05 was considered statistically significant. The workflow diagram of the study is depicted in Figure 1.

## 3. Results

DeepLabV3+, Mask R-CNN (Detectron2), YOLOv8, UNet, Super Vision UNet, and UNet Transformer were trained on 354 images and tested on 79 images. The scores of the six models for dental plaque segmentation on the test dataset are shown in Table 2. Among the six state-of-the-art models, UNet Transformer yielded the best results, with an IoU of 0.7845 and a Dice Coefficient of 0.8215. The optimum hyperparameters of the models are given in Table 3.

The prediction scores of the three dentists and the AI model (UNet Transformer) on 35 test images are summarized in Table 4, which demonstrates that the AI model achieves superior IoU scores compared to the three dentists. Although the AI model performed best in recall, Dice Coefficient, and IoU scores, it lagged behind the dentists in the precision score. The results of the *t*-tests are presented in Table 5. The AI model is effective, as evidenced by the IoU scores in Table 4 and by *t*-tests yielding *p*-values less than 0.05.

**Figure 2:**
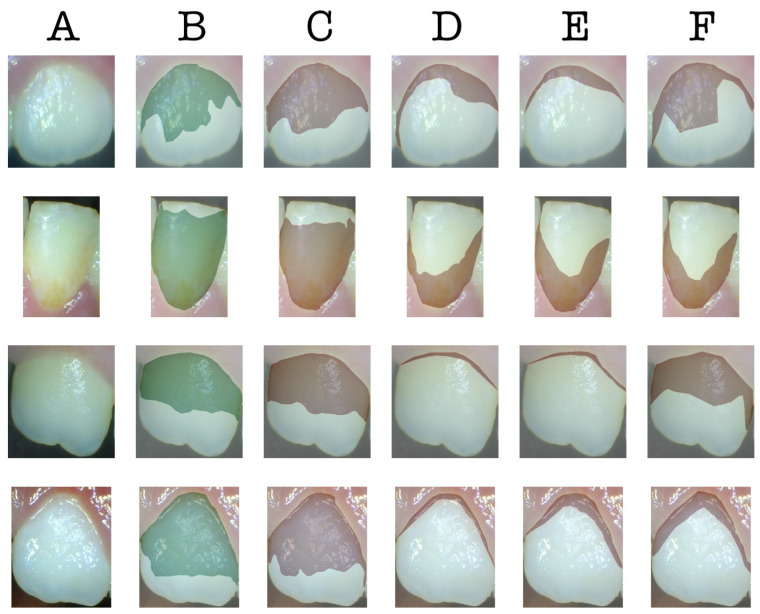
The samples of predictions. (**A**): input image, (**B**): ground truth, (**C**): predictions of the AI model (UNet Transformer), (**D**): predictions of dentist A, (**E**): predictions of dentist B, (**F**): predictions of dentist C.

**Figure 3:**
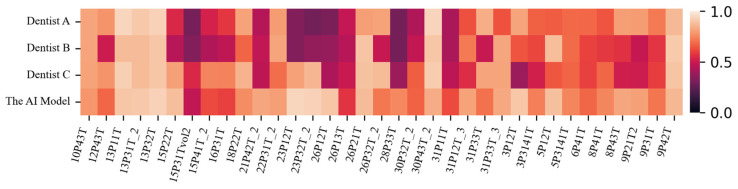
Heatmap visualization of IoU scores for the AI model and three dentists on the test dataset.

The sample images predicted by the AI model (UNet Transformer) and the three dentists, along with their corresponding ground truths, are depicted in Figure 2. According to this figure, the dental plaque predictions of the AI model are significantly closer to the ground truth compared to the predictions made by the three dentists. Figure 3 is the heatmap visualization of IoU scores for the AI model and three dentists on the test dataset. The color intensity represents the IoU values, where darker shades indicate lower scores and weaker segmentation agreement, while lighter shades correspond to higher IoU values and better agreement with the ground truth.

## 4. Discussion

Identifying dental plaque is essential to ensure that preventive and intervention treatments are safely provided to patients. Residual plaque can become more structured, making it harder to remove and more likely to harbor bacteria, which may contribute to oral diseases. If plaque is not properly eliminated, it can lead to gum inflammation (gingivitis), which may eventually progress to more severe conditions characterized by bone loss [6].

Identifying plaque in the young permanent teeth of children can be tricky for parents due to the similarity in color between the tooth surface and plaque. While staining the plaque with a disclosing agent helps visualize it, these agents have the potential to cause trauma in pediatric patients through changing the color of certain materials utilized in the restoration of young permanent teeth, resulting in the temporary staining of both the teeth, lips, tongue, hands, and clothes. The staining caused by disclosing agents may make individuals, especially children, feel self-conscious about their appearance, leading to discomfort in social settings. At the same time, these agents may provoke an unpleasant taste sensation in the oral cavity and elicit allergic reactions.

To overcome these limitations, AI-based dental plaque segmentation techniques offer a non-invasive, efficient, and automated alternative for plaque detection. Compared to conventional disclosing agents, AI-powered models, such as the UNet Transformer used in this study, provide a precise, real-time, and objective assessment of plaque presence without the need for staining agents. This approach eliminates patient discomfort, making plaque detection more accessible, especially in pediatric dentistry. Moreover, AI-assisted plaque detection can be highly effective for geriatric patients, individuals with motor impairments, and hospitalized patients, who often struggle with oral hygiene due to physical limitations [38,39]. This technology is particularly crucial for bedridden patients, as it helps prevent plaque buildup and the associated infections. Through telehealth and routine screenings, dentists can provide more personalized and effective care to these vulnerable populations.

Furthermore, traditional methods often rely on visual inspection by dentists, which is subjective and may vary based on expertise and lighting conditions. In contrast, AI-based models ensure consistent and standardized plaque detection, reducing the risk of human error. These advantages can enhance clinical workflow by reducing the time required for plaque assessment and supporting dentists in making more accurate treatment decisions. Additionally, integrating AI into routine dental examinations could help clinicians track changes in plaque accumulation over time, aiding in personalized preventive care and better patient education.

Several recent studies have explored deep learning architectures for dental plaque segmentation, each reporting varying degrees of success. Sudheera et al. attempted to detect dental plaque using Enhanced K-Means, which is a machine learning algorithm [14]. Imangaliyev et al. proposed a Convolutional Neural Network (CNN)-based deep learning model to classify dental red autofluorescence plaque on quantitative light-induced fluorescence (QLF) images, achieving an F1 score of 0.75 on the test dataset [15]. Liu et al. developed a Mask R-CNN-based dental health IoT platform to classify seven different oral diseases, including dental plaque [16]. Their platform has a 100% accuracy rate for dental plaque recognition, but not for segmentation. Li et al. presented a novel low-shot learning method for dental plaque segmentation using oral endoscope images [17]. It conducts low-shot learning at the super-pixel level, integrating local-to-global features for accurate pixel-level segmentation. The mIoU score of their model is 0.8585. In their subsequent work, they improved their model with a novel network featuring a self-attention module, achieving an mIoU score of 0.7364 [19]. You et al. utilized DeepLabV3+ for segmenting dental plaque in primary teeth and achieved a clinically acceptable mIoU of 0.726 compared to a pediatric dentist [18]. Similarly, Yüksel et al. applied DeepLabV3+ on intraoral images from pediatric patients, surpassing a dentist’s performance by achieving an IoU of 0.76, whereas the dentist achieved 0.71 [20]. Nantakeeratipat et al. focused on classifying plaque severity with Google Cloud Vertex AI AutoML, reporting 90.7% overall accuracy and notably high precision (98.3%) for the heavy plaque class [21]. Chen et al. combined YOLOv8 for tooth detection, the Segment Anything Model (SAM) for segmentation, and a CNN-based system called DeepPlaq for classification, achieving 94.1% accuracy in tooth detection and 84% in plaque classification [22]. Another approach is Song et al.’s CenterFormer, a transformer-based model integrating Cluster Center Encoder and Multiple Granularity Perceptions, which attained 60.91% IoU and 76.81% pixel accuracy, particularly benefiting low-contrast and variable plaque appearances [23].

An analysis of the table results indicates that UNet and its variants, namely Super Vision UNet and UNet Transformer, outperform other models in dental plaque segmentation. UNet Transformer achieves the best performance with an IoU of 0.7845 and a Dice Coefficient of 0.8215, demonstrating the effectiveness of Transformer-based approaches in segmentation tasks. While Super Vision UNet attains the highest recall, it falls behind UNet Transformer in terms of precision, reflecting the trade-off between detecting more plaque regions and accurately identifying them. The relatively lower performance of YOLOv8 may be attributed to its object-detection-based architecture, which may be less suited for segmentation tasks where capturing fine details is crucial. The lower precision of UNet Transformer suggests a tendency toward false positives, which could potentially be mitigated by increasing the number of healthy (plaque-free) samples in the dataset. Furthermore, UNet Transformer achieves higher IoU, recall, and Dice Coefficient values compared to measurements by dentists and, as indicated in Table 5, statistically outperforms three dentists. The visualization in Figure 3 highlights variations in segmentation performance across different cases, providing insights into the consistency and discrepancies between the AI model and human experts.

Our findings are consistent with previous studies in the literature, highlighting both the advantages and limitations of existing methods. While Transformer- and Unet-based models demonstrate superior performance compared to DeepLabV3+ models, it remains evident that further improvements are needed in dental plaque segmentation. Moreover, the results of this study align with recent research utilizing the O’Leary Plaque Score Index. For instance, Ramírez-Pedraza et al. categorized plaque accumulation into clinically interpretable levels, whereas this study enhances the accuracy and consistency of deep-learning-based plaque detection by ensuring alignment with established clinical standards [24].

The limitations of this study can be outlined as follows. First, although data augmentation techniques were applied to the training dataset, the overall number of images remained relatively small. Second, since the AI model was trained using images captured by a single type of camera, its performance with images from different camera brands and models remains uncertain. To address this limitation, domain adaptation techniques could be implemented alongside the inclusion of images taken with various camera brands. Third, due to the absence of a publicly available dental plaque dataset, a direct performance comparison with other models in the literature could not be conducted. Fourth, this study focused solely on predicting the presence of dental plaque; however, it did not assess plaque density, as the dataset lacked density information. Training the model with a dataset containing plaque density labels could enable the development of an AI system capable of predicting both the presence and density of plaque, potentially bringing AI-based methods closer to the gold standard in dental plaque assessment.

Although an intraoral camera with an integrated flash was used to capture images, ambient illumination was not further standardized with a studio flash before imaging. Variations in lighting conditions may have influenced image quality, potentially affecting the accuracy of plaque detection and model performance. While the intraoral camera provided a controlled imaging environment, differences in equipment and photographic techniques could still lead to variations in image color and resolution, impacting the reproducibility of results. Standardization of imaging was more feasible in the anterior region, as positioning teeth correctly before and after staining was easier in this area. However, achieving similar standardization in posterior teeth remains challenging. Future studies could explore the use of a stabilization device to ensure fixed-angle imaging for both anterior and posterior teeth. Further research is needed to refine this approach and validate its effectiveness.

## 5. Conclusions

This study presents an AI model for segmenting dental plaque in images of young permanent teeth. The proposed AI model demonstrates clinically superior performance in detecting and segmenting dental plaque compared to three experienced pediatric dentists. This finding highlights the potential of similar AI technologies to assist individuals in improving their oral health. Moreover, the superior performance of the AI model suggests its potential as a supportive tool for dentists, patients, and parents in accurately identifying dental plaque in future research. By training an AI model on images captured with a mobile device, individuals could conveniently detect plaque on their teeth using their smartphones, eliminating the need for specialized equipment. For future studies, training new AI models with a larger dataset containing images from various devices, along with density information of dental plaque, may lead to more accurate and precise results in plaque detection and segmentation.

## Figures and Tables

**Table 1:** Eligibility criteria: inclusion and exclusion factors.

Type	Criteria
Inclusion Criteria	Children aged 8–13 years
	Individuals in the mixed dentition phase
	Patients presenting at the Pediatric Dentistry Clinic, Hamidiye Faculty of Dental Medicine
	Patients randomly selected from those attending routine dental check-ups at a public hospital
	Individuals who were unaware of the study beforehand (i.e., had not received any prior oral health education or motivation)
Exclusion Criteria	Anterior young permanent teeth with enamel defects such as caries, hypoplasia, or hypomineralization
	Teeth with restorations or prosthetic treatments
	Young permanent teeth located in the posterior region
	Primary teeth

**Table 2:** Prediction scores of the AI models on the test dataset.

Model Name	Recall	Precision	Dice Coefficient	IoU
DeepLabV3+	0.7606	0.6664	0.7081	0.6575
Mask R-CNN (Detectron2)	0.8027	0.7471	0.7395	0.7229
Super Vison UNet	0.8277	0.8203	0.8240	0.7793
UNet	0.8095	0.8006	0.8037	0.7607
UNet Transformer	0.7718	0.8782	0.8215	0.7845
YOLOv8	0.5409	0.6600	0.5799	0.6157

**Table 3:** The optimum hyperparameters of the models.

Model Name	Image Size	Batch Size	Optimizer	Learning Rate
DeepLabV3+	192	2	Adam	$1\times 10^{-2}$
Mask R-CNN (Detectron2 with R50-DC5)	256	8	SGD	$1\times 10^{-2}$
Super Vision UNet	128	2	RMSProp	$1\times 10^{-4}$
UNet	192	4	Adam	$1\times 10^{-4}$
UNet Transformer	256	4	RMSProp	$1\times 10^{-4}$
YOLOv8	128	4	Adam	$1\times 10^{-4}$

**Table 4:** Prediction scores of the dentists and UNet Transformer on 35 test images.

	Recall	Precision	Dice Coefficient	IoU
Dentist A	0.5324	0.8661	0.6122	0.6565 ± 0.204
Dentist B	0.4405	0.8652	0.5304	0.6065 ± 0.196
Dentist C	0.6352	0.8494	0.6785	0.6898 ± 0.170
The AI model	0.7796	0.8398	0.7942	0.7783 ± 0.115

**Table 5:** *t*-test results comparing the AI model and the three dentists.

	t	df	*p*
AI model & Dentist A	−3.077	53.742	0.003
AI model & Dentist B	−4.467	55,009	0.000
AI model & Dentist C	−2.549	59.799	0.013

## Data Availability

Due to the sensitive nature of the data, the dataset used and analyzed during the current study can be made available from the corresponding author on reasonable request and after IRB approval has been obtained.

## Abbreviations

The following abbreviations are used in this manuscript:

AI	Artificial Intelligence
VGG	Visual Geometry Group
IoU	Intersection Over Union
